# Metronidazole Susceptibility Patterns in European *Bacteroides*, *Phocaeicola*, and *Parabacteroides* Clinical Isolates—A Systematic Review and Meta-Analysis

**DOI:** 10.3390/medsci14040424

**Published:** 2026-07-24

**Authors:** Kinga Markowska-Michalak, Anna Majewska, Hanna Pituch

**Affiliations:** Chair and Department of Medical Microbiology, Medical University of Warsaw, Chalubinskiego 5 Street., 02-004 Warsaw, Poland; kinga.markowska@wum.edu.pl (K.M.-M.); hanna.pituch@wum.edu.pl (H.P.)

**Keywords:** anaerobic bacteria, antimicrobial resistance, *Bacteroides*, *Phocaeicola*, *Parabacteroides*, metronidazole

## Abstract

**Objectives**: This study aimed to provide an updated overview of metronidazole susceptibility among human clinical isolates of *Bacteroides*, *Phocaeicola*, and *Parabacteroides* in Europe based on EUCAST breakpoint interpretation. **Methods**: A systematic review and meta-analysis were undertaken following searches of Web of Science, MEDLINE/PubMed, and Embase for studies published between 2011 and 2025. Eligible studies reported metronidazole susceptibility data, including minimum inhibitory concentrations and/or susceptible/resistant proportions, for clinical isolates from European countries. Risk of bias was assessed using criteria adapted from the Joanna Briggs Institute critical appraisal tool. Random-effects meta-analysis of proportions was used to estimate pooled susceptibility, with heterogeneity quantified by I^2^ and τ^2^; 95% prediction intervals and country subgroup analyses were also performed. Small-study effects were evaluated by funnel plot inspection, Egger’s and Begg’s tests. **Results**: Thirty-eight studies, comprising 72 country-specific datasets and 8501 isolates, were included. The pooled susceptibility proportion was 99.81% (95% CI 99.44–99.99%), with substantial heterogeneity (I^2^ = 68.2%; τ^2^ = 0.0038). The 95% prediction interval ranged from 95.75% to 100%. Although between-country differences were statistically significant, susceptibility remained consistently high overall. Lower values in Portugal and Switzerland came from single small datasets. **Conclusions**: These findings support the sustained in vitro activity of metronidazole across Europe, while highlighting the need for continued surveillance, particularly in underrepresented settings.

## 1. Introduction

Bacteroidales is an order of obligate anaerobic bacteria comprising numerous species that predominantly colonize the human gastrointestinal tract and represent some of the most abundant members of the gut microbiota. Within this order, the major clinically relevant taxa belong to the families Bacteroidaceae and Tannerellaceae, including the genera *Bacteroides*, the relatively recently reclassified genus *Phocaeicola*, and *Parabacteroides*. These bacteria often persist for extended periods within the human gut, where they undergo substantial evolutionary diversification through the accumulation of point mutations and the acquisition of prophages, mobile plasmids, and integrative conjugative elements [1,2]. *Bacteroides*, *Phocaeicola*, and *Parabacteroides* species are frequently isolated from intra-abdominal infections, including peritoneal infections, intra-abdominal abscesses, and postoperative wound infections, as well as from brain abscesses. Such infections are clinically challenging and frequently necessitate combined antimicrobial therapy together with surgical management. In women, these organisms play an important role in infections of the pelvic organs, including endometritis, tubo-ovarian abscesses, and postpartum infections. *Bacteroides fragilis* is an important opportunistic pathogen, and its antibiotic resistance has been investigated since the 1950s. Surveillance of resistance patterns in strains isolated from extraintestinal sites has continued over subsequent decades [3,4].

Antimicrobial resistance is an escalating public health challenge, and the environment—especially sewer networks, hospital and municipal wastewater, and wastewater treatment plants—is increasingly recognised as a source of resistant anaerobes, mainly the *B. fragilis* group (BFG), and antibiotic resistance genes (ARGs) [5]. Particularly important is resistance to metronidazole (MTZ), the first-line agent in the treatment of anaerobic infections. In Europe, the monitoring of antimicrobial resistance is guided by the European Committee on Antimicrobial Susceptibility Testing (EUCAST), which provides standardized interpretive criteria for susceptibility testing, thereby enabling the comparison of data generated across different clinical centres and countries. EUCAST published its first clinical breakpoints in 2008. It was subsequently implemented in virtually all European countries and has also been adopted by many countries outside Europe [6].

Anaerobic infections are most often managed empirically. Routine antimicrobial susceptibility testing (AST) of anaerobes is relatively uncommon in daily clinical practice, largely due to technical challenges, time requirements, and associated costs. Nevertheless, current recommendations emphasize that AST should be conducted in specific, well-defined clinical situations, such as severe or life-threatening infections, treatment failures, or infections caused by organisms isolated from sterile human body sites. In addition to its clinical relevance, periodic susceptibility testing is essential for epidemiological surveillance [7]. At present, several methodological approaches are available for the assessment of anaerobic antimicrobial susceptibility, including labour-intensive microdilution, gradient diffusion, and disc diffusion methods [8].

MTZ [1-(2-hydroxyethyl)-2-methyl-5-nitroimidazole] is a synthetic nitroimidazole antimicrobial agent that functions as a prodrug. MTZ enters the bacterial cell by passive diffusion and, under the low intracellular redox potential characteristic of Bacteroidales, undergoes reductive activation, resulting in the formation of a nitroradical anion (RNO_2_^•−^). Electron transfer to MTZ is mediated by low–redox potential electron transport proteins such as ferredoxin and flavodoxin, which are primarily maintained in a reduced state through pyruvate:ferredoxin oxidoreductase (PFOR)-dependent anaerobic metabolism. The resulting reactive intermediates primarily target DNA, causing DNA strand breakage and loss of DNA integrity, ultimately preventing DNA replication and transcription, leading to bacterial cell death. MTZ has an antimicrobial spectrum that includes obligate anaerobes such as *Bacteroides* and *Clostridium*, microaerophilic bacteria notably *Helicobacter pylori*, and protozoan parasites including *Giardia intestinalis* and *Trichomonas vaginalis*. MTZ has good oral bioavailability (>95%) and an elimination half-life of 5–10 h. It penetrates tissues well, reaches therapeutic concentrations in cerebrospinal fluid, and achieves high levels in brain abscesses. MTZ is approved by the Food and Drug Administration (FDA) for the treatment of various infections across multiple anatomical sites. The key indications for MTZ therapy, covering specific infection locations, include:

1. Intra-abdominal infections: peritonitis, abscesses, appendicitis, diverticulitis, and other infectious complications following abdominal surgery;
2. Bone and joint, skin and soft tissue infections including perioral skin infections and odontogenic abscesses;
3. Infections of the central nervous system (CNS); brain abscesses and other CNS infections caused by anaerobic bacteria;
4. Anaerobic pneumonia and lung abscesses when anaerobic infection is suspected or confirmed;
5. *Clostridioides difficile* infection (CDI), pseudomembranous colitis, particularly in mild to moderate cases;
6. Trichomoniasis, amebiasis, and giardiasis; a first-line therapy for infections caused by *T. vaginalis*, G. *intestinalis*, and *Entamoeba histolytica*;
7. *H. pylori* eradication;
8. Rosacea (topical therapy); therapeutic effects are related not to antibacterial properties but to potent anti-inflammatory and antioxidant activities.

MTZ is available in multiple formulations, including oral, intravenous, and topical dosage forms [9,10,11]. Understanding the FDA-approved indications for MTZ is essential for effective antibiotic stewardship and for preventing the development of antimicrobial resistance.

The literature provides substantial data on MTZ susceptibility among clinical anaerobes within the order Bacteroidales; however, direct comparisons across studies are often challenging due to heterogeneity in AST methods and—critically—differences in interpretive breakpoints. While many publications emphasize that MTZ resistance remains low, signals of an increase have been reported in selected settings. For example, the UK *Bacteroides* species surveillance survey comparing cohorts from 2000 and 2016 reported low absolute resistance rates but a ~6-fold proportional increase [12].

To our knowledge, this is the first systematic analysis focusing on clinically relevant anaerobic bacteria to synthesize MTZ susceptibility data for clinical isolates recovered from patients treated at healthcare centers across Europe. Antimicrobial susceptibility results were interpreted according to EUCAST clinical breakpoints, ensuring a standardized interpretive framework that minimized methodological variability and strengthened cross-study comparability.

This systematic review and meta-analysis aimed to assess temporal and geographical patterns of metronidazole susceptibility among *Bacteroides*, *Phocaeicola*, and *Parabacteroides* clinical isolates obtained from patients in Europe, based on studies published between 2011 and 2025.

## 2. Materials and Methods

### 2.1. Data Sources

This study was conducted as a systematic review and meta-analysis in accordance with PRISMA 2020 guidelines [13]. A comprehensive literature search was performed in three biomedical databases: MEDLINE/PubMed, Scopus, and Web of Science (WoS). Search strings included combinations of the following terms: *Bacteroides*, *Parabacteroides*, *Phocaeicola*, *metronidazole*, *resistance*, *susceptibility*, *drug resistance*, and *antimicrobial resistance*. Boolean operators (AND/OR) were applied, and the strategy was adapted to the indexing system of each database. The search was restricted to articles published in English within the time frame 2011–2025. In addition, bibliographic databases were screened manually to identify any relevant studies not retrieved during the initial search (Supplementary Table S1).

The research question was formulated using the PICO framework. The population comprised *Bacteroides*, *Phocaeicola*, and *Parabacteroides* clinical isolates recovered from human patients in European countries. The exposure of interest was time, operationalised as publication year (2011–2025). The outcomes included MTZ minimal inhibitory concentration (MIC) values and/or susceptibility categories (susceptible/resistant), interpreted exclusively according to EUCAST criteria. Eligible studies included observational studies, laboratory-based investigations, and epidemiological surveillance reports.

This systematic review and meta-analysis were not prospectively registered in the PROSPERO database.

### 2.2. Study Eligibility Criteria

The inclusion and exclusion criteria were defined *a priori* before the literature search and data extraction. Study selection was conducted in two stages. In the first phase, titles and abstracts were screened to remove irrelevant or clearly ineligible studies. In the second phase, the full texts of potentially relevant articles were assessed against the predefined criteria.

Inclusion criteria
Studies were included in the meta-analysis if they met all of the following criteria:

1. Study characteristics

- The study was conducted in Europe, and all bacterial isolates originated from European countries;
- The study was published between 2011 and 2025;
- The publication was written in English;
- The study was a peer-reviewed original research article.

2. Bacterial isolates

- The study included isolates belonging to the genera *Bacteroides*, *Parabacteroides*, or *Phocaeicola*;
- All isolates originated from human clinical samples;
- Species-level identification was performed using clearly described and accurate laboratory methods.

3. Antimicrobial susceptibility testing

- The study clearly specified the laboratory method used for metronidazole susceptibility testing (e.g., agar dilution, broth microdilution, gradient strip tests, disk diffusion);
- The study reported either MIC values and/or proportions of susceptible/resistant isolates for metronidazole;
- AST results were interpreted according to EUCAST recommendations, or the study provided MIC values enabling reinterpretation based on EUCAST breakpoints;
- AST results were not pooled with other taxa.

Exclusion criteria
Studies were excluded if any of the following conditions were met:

- No EUCAST-based (e.g., CLSI; Clinical & Laboratory Standards Institute) interpretation of metronidazole susceptibility or no possibility to map MIC values to EUCAST breakpoints;
- Inclusion of animal, environmental, or non-European isolates;
- Absence of MIC values or metronidazole susceptibility/resistance proportions;
- Case reports, conference abstracts, reviews, or studies lacking quantitative susceptibility data.

### 2.3. Data Extraction

Data extraction was carried out independently by two reviewers using a predefined template. All extracted data were entered into a structured Microsoft Excel worksheet. Information collected included the study location and period, the number of isolates, species names, methods used for species identification, the AST method, interpretive criteria, MIC values, and the proportions of susceptible or resistant isolates. Only studies that allowed susceptibility assessment according to EUCAST breakpoints—either by applying EUCAST guidelines or by providing MIC data suitable for EUCAST reinterpretation—were included in the quantitative analyses.

### 2.4. Assessment of Risk of Bias

The risk of bias was assessed using nine criteria adapted from the Joanna Briggs Institute (JBI) critical appraisal tool for prevalence studies [14]. These criteria covered aspects of both internal and external validity and were applied with equal weight (Supplementary Table S2). The initial assessment was conducted by one reviewer and subsequently cross-checked by two independent reviewers. Based on the number of criteria fulfilled, studies were categorized as having low (7–9 criteria met), moderate (4–6), or high (1–3) risk of bias.

### 2.5. Methods of Data Synthesis

A meta-analysis of proportions was conducted to estimate the pooled proportion of MTZ-susceptible *Bacteroides* spp., *Phocaeicola* spp., and *Parabacteroides* spp. isolates using the metaprop function from the meta package in R (R Foundation for Statistical Computing, Vienna, Austria). A random-effects model was applied, with between-study variance (τ^2^) estimated using restricted maximum likelihood (REML). Proportions were transformed using the Freeman-Tukey double arcsine method. In the main analysis, when studies reported data from multiple countries, each country-specific dataset was treated as a separate analytical entry. Heterogeneity was assessed using Cochran’s Q test and quantified using the I^2^ statistic and τ^2^. A 95% prediction interval was calculated to estimate the expected range of susceptibility. Potentially influential analytical entries were assessed using influence diagnostics in the metafor package. Small-study effects were evaluated using publication-level funnel plots and Egger’s and Begg’s tests; for these analyses, multicountry studies were aggregated at the publication level to avoid overrepresentation. Subgroup analyses were conducted by country to explore geographical variation. Meta-regression by time was not performed because studies analyzed isolates collected over different and often broad time periods, which were inconsistently reported, making publication year an invalid proxy for the actual period of isolate collection or testing. Geographical patterns were visualized using proportional symbol maps.

### 2.6. Study Selection

**Figure 1:**
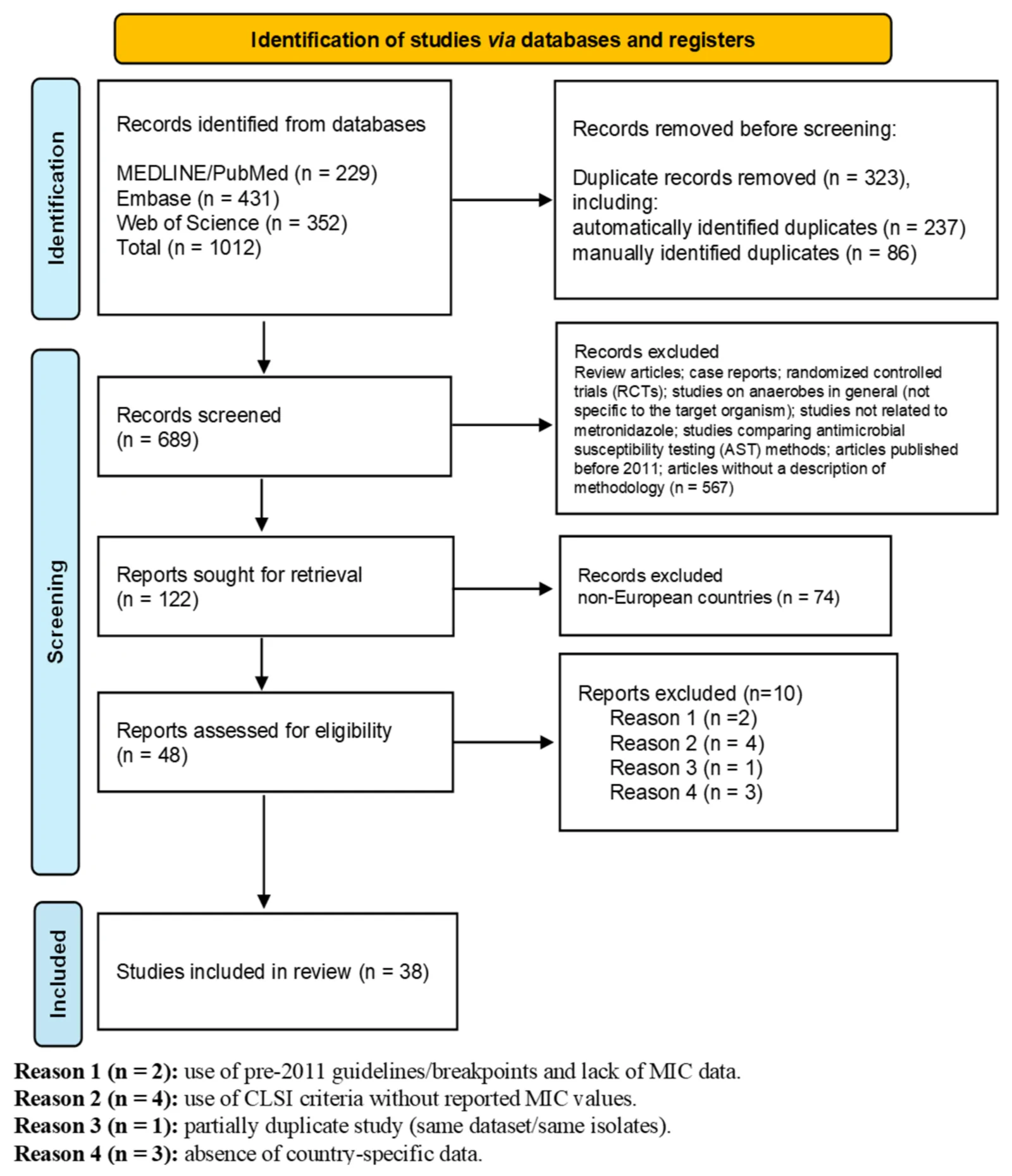
PRISMA flow diagram of study selection.

A total of 1012 records were identified through the systematic search and imported into the reference manager software (EndNote v. 21). After removal of 323 duplicates, the remaining records underwent screening. Following title/abstract screening and full-text assessment, 38 studies met the inclusion criteria and were included in the quantitative synthesis (Figure 1) [3,12,15,16,17,18,19,20,21,22,23,24,25,26,27,28,29,30,31,32,33,34,35,36,37,38,39,40,41,42,43,44,45,46,47,48,49,50]. The characteristics of the included studies and the extracted data are provided in Supplementary Table S3.

### 2.7. Characteristics of the Evidence Base

The 38 included studies were published between 2011 and 2025 and contributed 72 country-specific datasets from 23 European countries: Austria, Belgium, Croatia, the Czech Republic, Denmark, Estonia, Finland, France, Germany, Greece, Hungary, Italy, the Netherlands, Poland, Portugal, Romania, the Russian Federation, Slovenia, Spain, Sweden, Switzerland, Turkey, and the United Kingdom. Overall, 8501 isolates of *Bacteroides* spp., *Phocaeicola* spp., and *Parabacteroides* spp. were included, with 132 isolates (1.55%) classified as resistant. Isolate collection/testing years ranged from 2000 to 2023. Most studies were single-country studies (34/38), whereas four provided multicountry data and were split into separate country-specific analytical entries for the main meta-analysis. The median dataset size was 73.5 isolates (IQR: 31–120.5; range: 3–869). Sixteen countries were represented by at least two datasets, and 14 by at least three datasets.

### 2.8. Risk of Bias in Included Studies

According to the criteria adapted from the Joanna Briggs Institute (JBI), 37 studies were classified as low risk of bias, one as moderate risk, and none as high risk. The most frequent methodological limitations were related to isolate selection (23 studies did not meet this criterion, due to a lack of information on whether isolates originated from structured epidemiological surveillance programmes or represented consecutive isolates collected during routine microbiological diagnostics), followed by inadequate description of the study population and setting (6 studies), and issues with the validity of condition identification and laboratory procedure quality (3 studies each). Less frequent limitations included irrelevant statistical analysis (2 studies) and lack of disclosure of conflicts of interest or funding (2 studies). In contrast, sample representativeness, adequacy of sample size, reporting of results, handling of missing data, and standardization of measurement were generally well addressed across the included studies.

## 3. Results

### 3.1. Overall Meta-Analysis

**Figure 2:**
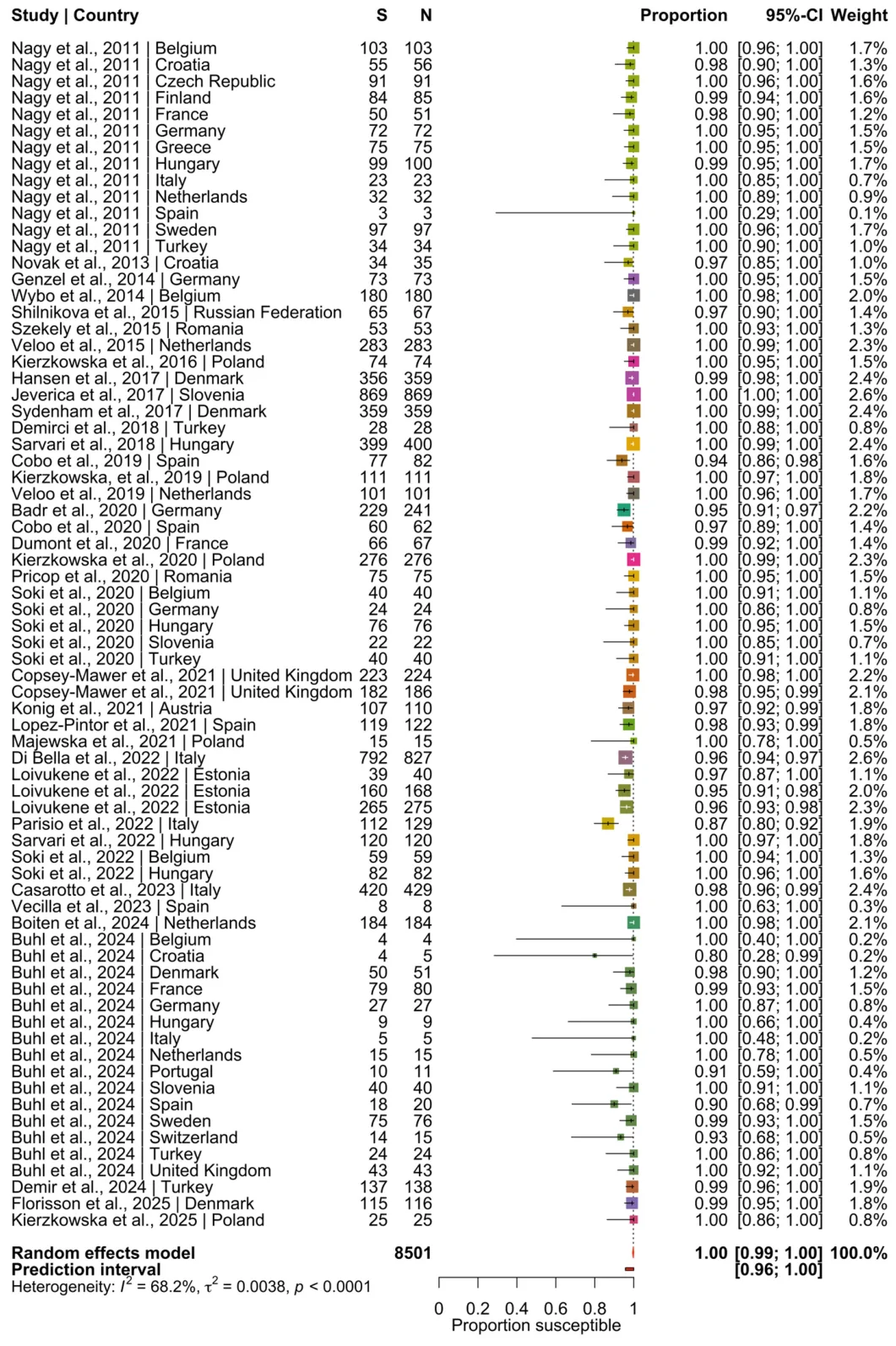
Forest plot of pooled susceptibility of *Bacteroides*, *Parabacteroides*, and *Phocaeicola* to MTZ. Estimates are shown with 95% confidence intervals; the pooled estimate was calculated using a random-effects model. Datasets from the same publication are indicated by identically coloured squares. Legend: S; susceptibility, N; number of isolates, heterogeneity was moderate to substantial (I^2^ = 68.2%; τ^2^ = 0.0038).

The pooled MTZ susceptibility proportion estimated using a random-effects model was 99.81% (95% CI: 99.44–99.99%). Substantial heterogeneity between datasets was observed (Q = 223.15, df = 71, *p* < 0.001), with an I^2^ value of 68.2% (95% CI: 59.4–75.0%) and between-study variance τ^2^ = 0.0038. The 95% prediction interval ranged from 95.75% to 100%, indicating that susceptibility in comparable settings is expected to remain very high. The forest plot summarizing individual dataset estimates and the pooled susceptibility proportion is shown in Figure 2.

### 3.2. Heterogeneity, Influence, and Robustness Analyses

The Baujat plot indicated that heterogeneity was driven by a small number of datasets, with Parisio et al. [39] standing out most clearly. Several others, including Badr et al. [15], Di Bella et al. [23], Cobo et al. [20], Loivukene et al. [34], and Jeverica et al. [29], showed more moderate influence. Excluding Parisio et al. [39] slightly increased the pooled estimate to 99.89% (95% CI: 99.60–100%) and reduced τ^2^ to 0.0027 but did not substantively change the overall conclusion (Supplementary Table S4 and Supplementary Figure S1).

Only six of the 72 datasets showed susceptibility below 95%. Four of these arose from the multicountry dataset of Buhl et al. [17] and were based on small sample sizes (Croatia, *n* = 5; Portugal, *n* = 11; Spain, *n* = 20; Switzerland, *n* = 15). The remaining two were Cobo et al. [20] from Spain (77/82 MTZ susceptible; 93.9%) and Parisio et al. [39] from Italy (112/129 MTZ susceptible; 86.8%).

### 3.3. Country-Specific Subgroup Analyses

**Figure 3:**
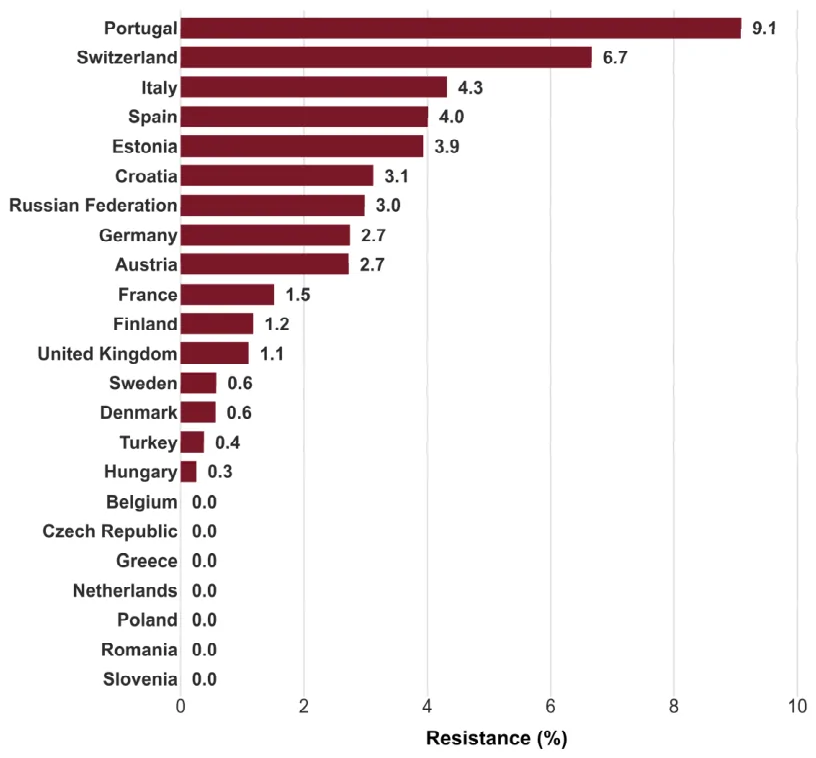
MTZ resistance rates by country based on meta-analysis of proportions. Values represent pooled resistance percentages (R/N × 100) derived from logit-transformed models and ordered by resistance level.

Subgroup meta-analysis stratified by country demonstrated significant differences in susceptibility across countries (Q = 104.38, df = 22, *p* < 0.001). Most country-specific pooled estimates remained close to 100%, often with narrow confidence intervals (Figure 3). Within-country heterogeneity was low in many subgroups; among countries represented by at least two datasets, 10 of 16 showed I^2^ = 0%, indicating high consistency across studies. Notable exceptions were Italy (I^2^ = 81.36%), Germany (I^2^ = 65.05%), and Denmark (I^2^ = 54.69%), where substantial within-country variability was observed. Among countries represented by more than one dataset, the lowest pooled MTZ susceptibility estimates were observed in Estonia (96.32%) and Italy (97.30%) (Figure S2).

### 3.4. Geographic Distribution and Country-Level Totals

**Figure 4:**
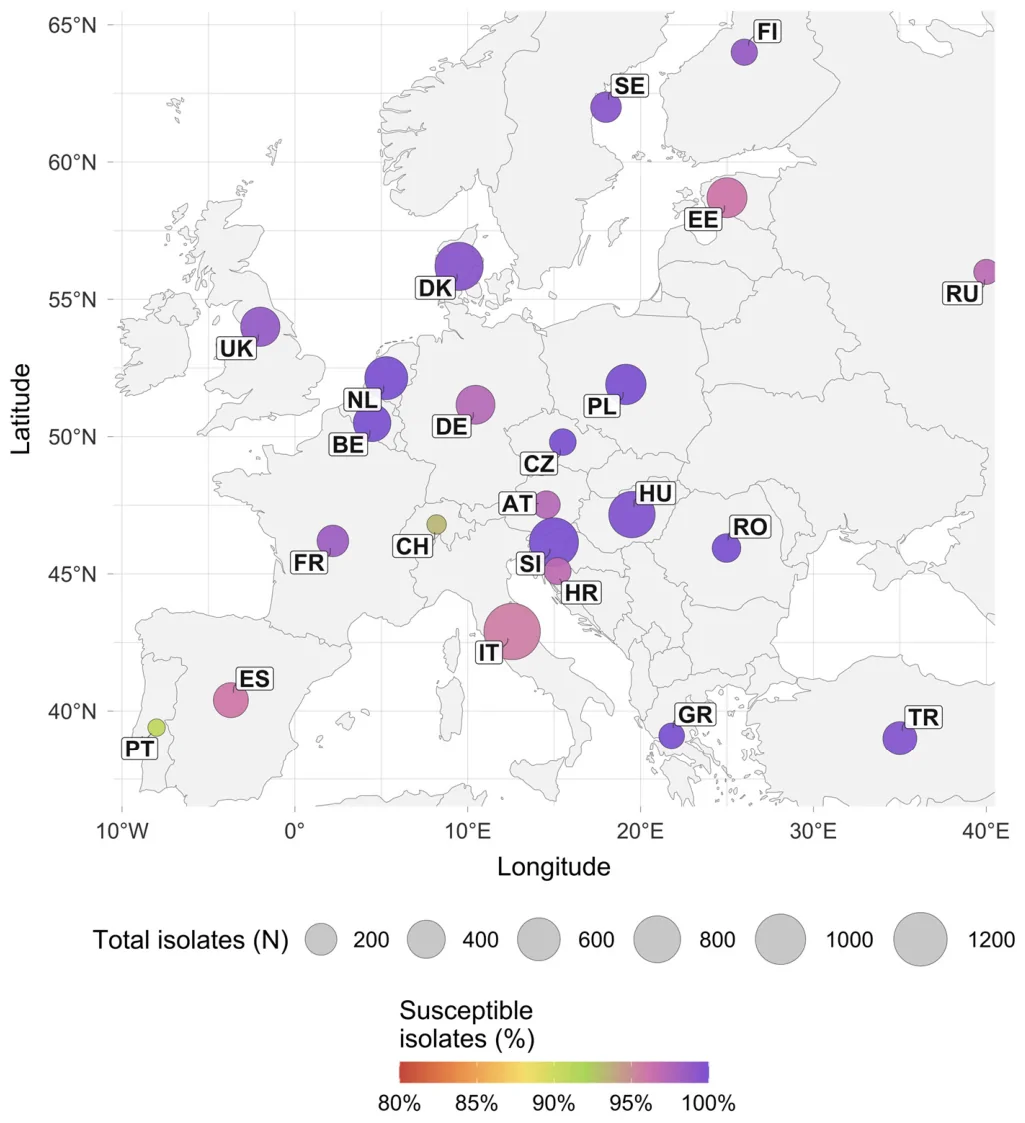
Geographic distribution of *Bacteroides*, *Phocaeicola*, and *Parabacteroides* isolates in Europe. Bubble size represents the number of isolates per dataset; colour indicates the proportion susceptible to MTZ. Legend: AT, Austria; BE, Belgium; CH, Switzerland; CZ, Czech Republic; DE, Germany; DK, Denmark; EE, Estonia; ES, Spain; FI, Finland; FR, France; GR, Greece; HR, Croatia; HU, Hungary; IT, Italy; NL, the Netherlands; PL, Poland; PT, Portugal; RO, Romania; RU, Russian Federation; SE, Sweden; SI, Slovenia; TR, Turkey; UK, United Kingdom.

The geographic distribution of MTZ susceptibility across Europe is presented in Figure 4. The largest numbers of isolates were reported from Italy (*n* = 1413), Slovenia (*n* = 931), Denmark (*n* = 885), Hungary (*n* = 787), and the Netherlands (*n* = 615), which together accounted for 54.5% of all included isolates. When country-level data were aggregated descriptively, susceptibility was 100% in 7 of 23 countries and at least 95% in 21 of 23 countries.

The lowest aggregated country-level susceptibility values were observed in Portugal (90.91%), Switzerland (93.33%), Italy (95.68%), and Spain (95.96%) (Figure 3). These estimates should be interpreted cautiously, particularly for Portugal and Switzerland, which were each represented by a single small dataset (*n* = 11 and *n* = 15, respectively). Resistant isolates were concentrated disproportionately in a small number of countries: Italy, Estonia, Germany, and Spain together accounted for 78.8% of all resistant isolates identified in the dataset.

### 3.5. Small-Study Effects and Publication Bias

**Figure 5:**
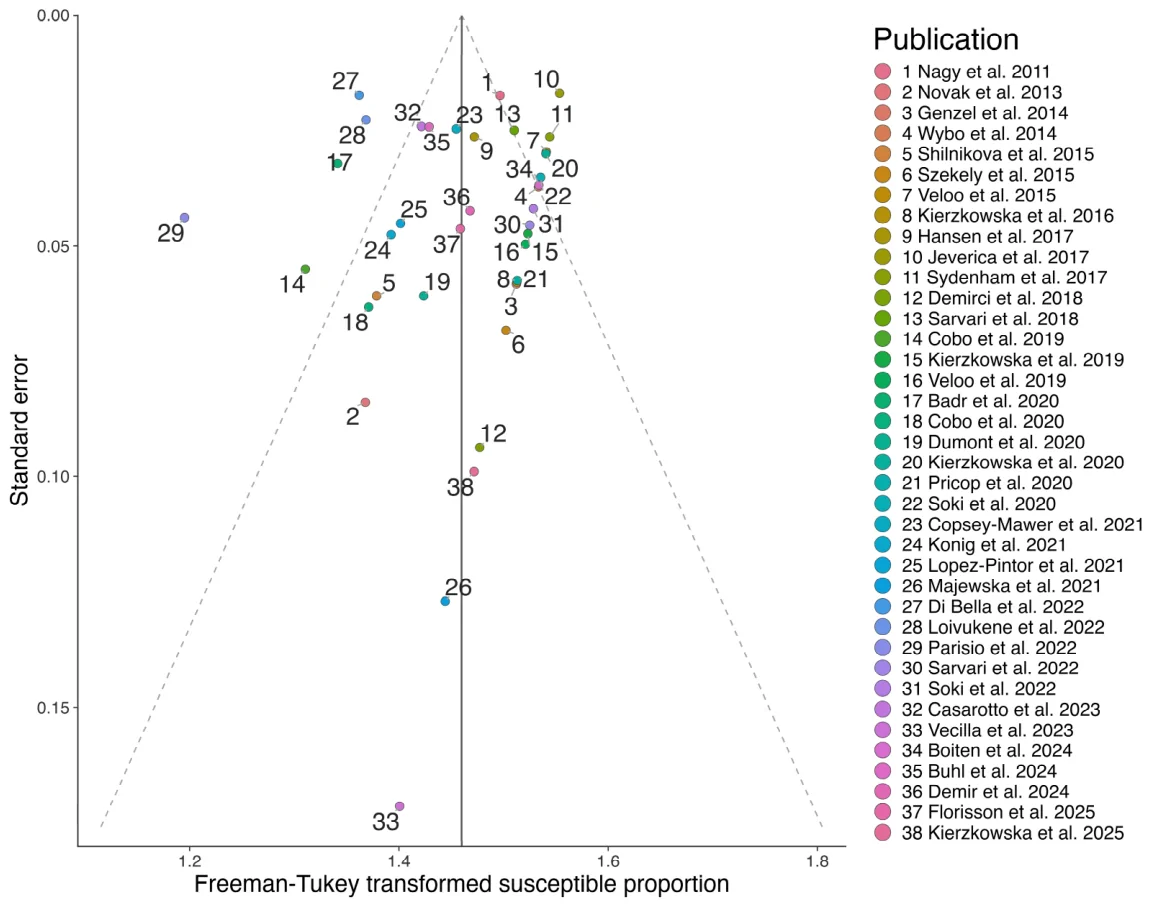
Funnel plot of publication-level susceptibility estimates.

Publication-level funnel plot (Figure 5) suggested asymmetry, with fourteen studies lying outside the 95% pseudo-confidence limits: Nagy et al. [37], Wybo et al. [50], Veloo et al. [48], Jeverica et al. [29], Sydenham et al. [46], Sarvari et al. [42], Cobo et al. [20], Badr et al. [15], Kierzkowska et al. [30], Soki et al. [44], Di Bella et al. [23], Loivukene et al. [34], Parisio et al. [39], and Boiten et al. [16]. Of these, nine lay to the right and five to the left of the 95% pseudo-confidence limits. However, Egger’s regression test did not indicate significant funnel plot asymmetry (t = −0.42, df = 36, *p* = 0.68). Similarly, Begg’s rank correlation test was not statistically significant (z = −1.52, *p* = 0.13). Taken together, these findings did not provide statistically significant evidence of small-study effects or publication bias.

## 4. Discussion

Interpretation of AST results relies primarily on two internationally recognized breakpoint systems: the EUCAST and CLSI, which are not always consistent in terms of methodology, breakpoints, and testing recommendations [51]. CLSI is predominantly used in the United States and many regions outside Europe, whereas EUCAST is the preferred system in Europe. Advantages of EUCAST include a more transparent and rigorous breakpoint-setting process, the absence of formal industry representation, and freely accessible guidelines. In contrast, CLSI documents require an annual purchase (only selected breakpoint data are available online) [6,52].

Interpretative criteria for MTZ against *Bacteroides* spp. differ markedly between EUCAST and CLSI. EUCAST defines resistance as an MIC > 4 mg/L and, using disk diffusion with a 5 μg disk, susceptibility as a zone diameter ≥ 25 mm. CLSI defines resistance as an MIC ≥ 32 mg/L. As a result, an isolate with an MIC of 8 mg/L would be categorized as susceptible (non-resistant) according to CLSI but resistant according to EUCAST [37,53]. Furthermore, EUCAST explicitly states that the breakpoints established for *Bacteroides* spp. are also applicable to *Parabacteroides* spp. and *Phocaeicola* spp., including *Ph. dorei* and *Ph. vulgatus* [53]. For anaerobes, EUCAST/CLSI differences are not confined to breakpoint thresholds but also reflect methodological variation (e.g., media composition and incubation conditions), which may influence MIC determinations and thus susceptibility categorisation.

The first *B. fragilis* strain resistant to MTZ was reported in 1978. Resistance to this antibiotic in *Bacteroides* spp. is primarily associated with the acquisition of *nim* genes (e.g., *nimA–nimL*), which encode proteins with nitroimidazole reductase activity; additional proposed mechanisms include reduced activity of pyruvate:ferredoxin oxidoreductase and other electron-transfer systems involved in drug activation, enhanced DNA repair systems counteracting MTZ-induced DNA damage, increased oxygen tolerance, and, less consistently, efflux-mediated decreases in intracellular drug accumulation [54,55].

This study indicates that MTZ susceptibility among *Bacteroides*, *Phocaeicola*, and *Parabacteroides* clinical isolates collected in Europe remains high, with a pooled susceptibility estimate of 99.81% (95% CI: 99.44–99.99%). These findings indicate preserved in vitro activity of MTZ against these organisms across Europe. Although moderate-to-substantial between-dataset heterogeneity was observed, susceptibility remained high across countries overall. Some of the most extreme descriptive country-level values were influenced by small denominators; for instance, the estimate for Portugal (90.91%) was derived from 10 of 11 tested isolates, compared with 785 of 787 isolates underlying the estimate for Hungary (99.75%). This illustrates how a single non-susceptible isolate can markedly affect percentage estimates when sample sizes are very small. At the same time, not all between-country variation can be attributed to sample size alone, as subgroup analysis showed statistically significant differences across countries and substantial within-country heterogeneity in Italy, Denmark, and Germany. The overall conclusion was robust in influence analyses: exclusion of the most influential dataset, Parisio et al. [39], slightly increased the pooled estimate to 99.89% and reduced τ^2^ to 0.0027 but did not significantly alter the interpretation.

Important information on the antimicrobial susceptibility patterns of anaerobic bacteria in Europe is provided by the ANAEuROBE study. This large multicentre retrospective surveillance study analysed 14,527 anaerobic isolates recovered from blood cultures collected in 44 European hospital centres across 22 countries between 2020 and 2023. The ANAEuROBE study demonstrated the presence of antibiotic resistance among anaerobic species, reporting MTZ resistance rates, based on MIC determination according to EUCAST criteria, of 5% for *Ph. vulgatus*, 4% for *B. uniformis*, 3% for *B. fragilis* and *B. thetaiotaomicron*, 2% for *B. ovatus*, and 1% for *P. distasonis*. However, the ANAEuROBE study was not included in the quantitative synthesis of the present meta-analysis because the available data did not allow precise extraction of the information required for statistical analysis. In addition to AST data, the authors conducted a survey among participating microbiologists, revealing broad recognition of the clinical importance of anaerobic infections but substantial variability in diagnostic capacity and routine AST practices. These findings reinforce the need for continuous surveillance of antimicrobial resistance trends to optimise empirical therapy and inform future treatment guidelines [56].

MTZ, therefore, remains highly active against *Bacteroides* spp. and closely related species, supporting its role as a first-line option for the treatment of many anaerobic infections. This situation contrasts markedly with that observed in *H. pylori*, where resistance to MTZ is common and represents a growing clinical challenge. In a large global analysis including 63 studies and 15,953 individuals from 28 countries across five World Health Organization (WHO) regions, primary resistance of *H. pylori* to MTZ was high, with a pooled resistance rate of 35.3%, highlighting its persistent and widespread clinical significance [57]. A retrospective study of over 21,000 patients in Europe showed that the rate of MTZ resistance was around 32% [58]. Consequently, MTZ has remained a cornerstone of therapy for infections caused by *Bacteroides* spp. despite long-term use.

The strengths of this study include the use of an EUCAST-based interpretation across all included studies, which ensured methodological consistency and enhanced the comparability of results. Focusing exclusively on European studies reduced epidemiological heterogeneity and increased the clinical relevance of the findings for this region.

A limitation of this study is the geographical coverage of the available data, as the included studies represent several European countries, while some regions remain underrepresented or are not represented.

## 5. Conclusions

Among *Bacteroides*, *Phocaeicola*, and *Parabacteroides* species isolated from patients treated in healthcare facilities across European countries (2000–2023), phenotypic susceptibility to MTZ remains consistently high. Nevertheless, sustained monitoring across Europe, together with complementary regional initiatives, is essential to detect early shifts in susceptibility patterns and to support timely clinical and public health responses.

## Data Availability

The original contributions presented in this study are included in the article/Supplementary Materials. Further inquiries can be directed to the corresponding author.

## Supplementary Materials

The following supporting information can be downloaded at https://www.mdpi.com/article/10.3390/medsci14040424/s1, Table S1: Search strategy. Table S2: Criteria used for risk of bias assessment; Table S3: Study characteristics and extracted data. Table S4: Funnel plot analysis identifying publication-level estimates lying outside the 95% funnel boundaries. Figure S1: Baujat plot based on publication-level data. Figure S2: Forest plot of susceptibility proportions by study and country (random-effects model). File S1: PRISMA Checklist.

## Abbreviations

The following abbreviations are used in this manuscript:

BFG	*Bacteroides fragilis* Group
ARGs	Antibiotic Resistance Genes
MTZ	Metronidazole
EUCAST	European Committee on Antimicrobial Susceptibility Testing
AST	Antimicrobial Susceptibility Testing
PFOR	Pyruvate: Ferredoxin Oxidoreductase
DNA	Deoxyribonucleic Acid
FDA	Food and Drug Administration
CNS	Central Nervous System
CDI	*Clostridioides Difficile* Infection
PRISMA	Preferred Reporting Items for Systematic Reviews and Meta-Analyses
WoS	Web of Science
PICO	Patient (Problem, or Population), Intervention, Comparison (Control, or Comparator), Outcome(s)
MIC	Minimum Inhibitory Concentration
CLSI	Clinical and Laboratory Standards Institute
JBI	Joanna Briggs Institute
REML	Restricted Maximum Likelihood
IQR	Interquartile range
AT	Austria
BE	Belgium
CH	Switzerland
CZ	Czech Republic
DE	Germany
DK	Denmark
EE	Estonia
ES	Spain
FI	Finland
FR	France
GR	Greece
HR	Croatia
HU	Hungary
IT	Italy
NL	Netherlands
PL	Poland
PT	Portugal
RO	Romania
RU	Russian Federation
SE	Sweden
SI	Slovenia
TR	Turkey
UK	United Kingdom
WHO	World Health Organization
