# Supplementary material for: Metronidazole Susceptibility Patterns in European Bacteroides, Phocaeicola, and Parabacteroides Clinical Isolates—A Systematic Review and Meta-Analysis
Source: Med Sci (Basel). 2026 Jul 24;14(4):424. doi: 10.3390/medsci14040424 (PMC13515583; doi:10.3390/medsci14040424)
Supplement: Supplementary file 1 [file medsci-14-00424-s001.zip › Supplementary Figures and Tables.pdf]

medical sciences

MDPI

Metronidazole Susceptibility Patterns in European *Bacteroides, Phocaeicola,* and *Parabacteroides* Clinical Isolates—A Systematic Review and Meta-Analysis

Table S1. Search strategy.

Database	Search date	Years covered	Full electronic search strategy	Filters	Results
Medline/PubMed	March 1, 2026	2011–2025	(Bacteroides OR Parabacteroides OR Phocaeicola) AND (metronidazole) AND (resistance OR susceptibility OR "drug resistance" OR "antimicrobial resistance") AND ("2011/01/01"[PDAT]: "2025/12/31"[PDAT])	English Humans	229
Embase	March 1, 2026	2011–2025	(bacteroides OR parabacteroides OR phocaeicola) AND metronidazole AND (resistance OR susceptibility OR 'drug resistance' OR 'antimicrobial resistance') AND [2011-2025]/py AND [english]/lim AND [humans]/lim AND [embase]/lim	Humans, English, Clinical study, Years 2011–2025	431
Web of Science	March 1, 2026	2011–2025	TS = (Bacteroides OR Parabacteroides OR Phocaeicola) AND TS = (metronidazole) AND TS = (resistance OR susceptibility OR "drug resistance" OR "antimicrobial resistance")	Year filter 2011–2025	352

Table S2. Criteria used for risk of bias assessment.

**Criteria used for the methodological quality assessment**	
1. Sample representativeness	Is the sample frame appropriate and clearly defined for the target population of clinical isolates?
2. Isolate selection	Was the method of isolate inclusion appropriate and clearly described?
3. Adequacy of sample size	Was the number of isolates sufficient to reliably estimate proportions?
4. Description of study population and setting	Were the studied isolates and setting described in sufficient detail?
5. Validity of condition identification	Were valid methods used for bacterial identification?
6. Standardization of measurement	Was susceptibility/resistance measured in a standardized and comparable way for all isolates?
7. Laboratory procedure quality	Was the laboratory procedure described in sufficient detail?
8. Reporting of results and missing data	Were the numerator and denominator clearly reported, and were missing/excluded data explained?
9. Appropriateness of statistical analysis	Was the analysis appropriate for proportional data?
10. Conflicts of interest/funding	Were funding sources and conflicts of interest clearly disclosed?

Table S3. Study characteristics and extracted data.

Reference	Author	Year of testing	Country	Number of isolates	Susceptibility [%]	Resistance [%]	Resistant [N]	Susceptible [N]	Criteria fulfilled (according to Table S2)	Risk of bias
[37]	Nagy *et al*., 2011	2008-2009	Finland	85	98.8	1.2	1	84	8/10 (1,3,4,6-10)	Low
			Sweden	97	100	0	0	97		
			Netherlands	32	100	0	0	32		
			Belgium	103	100	0	0	103		
			Germany	72	100	0	0	72		
			Spain	3	100	0	0	3		
			France	51	98	2	1	50		
			Greece	75	100	0	0	75		
			Italy	23	100	0	0	23		
			Turkiye	34	100	0	0	34		
			Croatia	56	98.2	1.8	1	55		
			Czech Republic	91	100	0	0	91		
			Hungary	100	99	1	1	99		
[38]	Novak *et al*., 2013	2013	Croatia	35	97.1	2.9	1	34	9/10 (1,3-10)	Low
[27]	Genzel *et al*., 2014	2008-2009	Germany	73	100	0	0	73	7/10 (1,3,6-10)	Low
[50]	Wybo *et al*., 2014	2011-2012	Belgium	180	100	0	0	180	9/10 (1,3-10)	Low
[43]	Shilnikova *et al*. 2015	2004-2014	Russian Federation	67	97	3	2	65	8/10 (1, 3-8,10)	Low
[47]	Szekely *et al*., 2015	2010-2013	Romania	53	100	0	0	53	9/10 (1-3,5-10)	Low
[48]	Veloo *et al*., 2015	2011-2013	Netherlands	*283*	100	0	0	283	8/10 (1,3,5-10)	Low
[31]	Kierzkowska *et al*., 2016	2013-2015	Poland	74	100	0	0	74	9/10 (1,3-10)	Low
[28]	Hansen *et al*., 2017	2014-2015	Denmark	359	99.2	0.8	3	356	9/10 (1,3-10)	Low
[46]	Sydenham *et al*., 2017	2009-2012	Denmark	359	100	0	0	359	9/10 (1,3-10)	Low
[29]	Jeverica *et al*. 2017	2015	Slovenia	869	100	0	0	869	9/10 (1,3-10)	Low
[22]	Demirci *et al*., 2018	2004-2014	Turkey	28	100	0	0	28	8/10 (1,3,4,6-10)	Low
[42]	Sarvari *et al*., 2018	2014-2016	Hungary	400	99.75	0.25	1	399	8/10 (1,3,5-10)	Low
[20]	Cobo *et al*., 2019	2017-2018	Spain	82	94	6	5	77	8/10 (1,3,5-10)	Low
[32]	Kierzkowska, *et al*., 2019	2007-2012	Poland	111	100	0	0	111	8/10 (1,3-8,10)	Low
[49]	Veloo *et al*., 2019	2016	Denmark	101	100	0	0	101	10/10	Low
[15]	Badr *et al*., 2020	2015-2019	Germany	368	96.2	3.8	14	354	9/10 (1,3-10)	Low
[24]	Dumont *et al*., 2020	2015-2016	France	67	98.5	1.5	1	66	9/10 (1-6,8-10)	Low
[30]	Kierzkowska *et al*., 2020	2013-2017	Poland	276	100	0	0	276	8/10 (1,3-9)	Low
[40]	Pricop *et al*., 2020	2015-2016	Romania	75	100	0	0	75	9/10 (1,3-10)	Low
[44]	Soki *et al*., 2020	2014-2016	Belgium	40	100	0	0	40	8/10 (1,3,5-10)	Low
			Germany	24	100	0	0	24		
			Hungary	76	100	0	0	76		
			Slovenia	22	100	0	0	22		
			Turkey	40	100	0	0	40		
[12]	Copsey-Mawer, *et al*., 2021	2000	United Kingdom	224	99.6	0.4	1	223	10/10	Low
		2016	United Kingdom	168	97.6	2.3	4	162		
[19]	Cobo *et al*., 2020	2016-2020	Spain	62	96.8	3.2	2	60	10/10	Low
[33]	König *et al*., 2021	2019	Austria	110	97.3	2.7	3	107	9/10 (1,3-10)	Low
[35]	Lopez-Pintor *et al*., 2021	2018-2019	Spain	122	97.5	2.5	3	119	10/10	Low
[36]	Majewska *et al*., 2021	2013–2020	Poland	15	100	0	0	15	10/10	Low
[23]	Di Bella *et al*., 2022	2016-2020	Italy	827	95.8	4.2	35	792	10/10	Low
[34]	Lõivukene *et al*., 2022	2021	Estonia	275	96.4	3.6	10	265	10/10	Low
		2016	Estonia	160	100	0	0	160		
		2010	Estonia	39	97.5	2.5	1	39		
[39]	Parisio *et al*., 2022	2019	Italy	129	94.6	5.4	17	112	9/10 (1-9)	Low

[41]	Sarvari *et al*., 2022	2004-2012	Hungary	120	100	0	0	120	10/10	Low
[45]	Soki *et al*., 2022	2014-2016	Belgium	59	100	0	0	59	6/10 (1,3,4,8-10)	Moderate
			Hungary	82	100	0	0	82		
[18]	Casarotto *et al*., 2023	2019-2021	Italy	429	97.9	2.1	9	420	9/10 (1,3-10)	Low
[25]	Fernández Vecilla *et al*., 2023	2010-2023	Spain	8	100	0	0	8	9/10 (1,2,4-10)	Low
[16]	Boiten *et al.*, 2024	2021	Netherlands	184	100	0	0	184	8/10 (1,3-9)	Low
[17]	Buhl *et al*., 2024	2022	Belgium	4	100	0	0	4	10/10	Low
			Croatia	5	80	20	1	4		
			Denmark	51	96	4	1	49		
			France	80	98.75	0.25	1	79		
			Germany	27	100	0	0	27		
			Hungary	9	100	0	0	9		
			Italy	5	100	0	0	5		
			Netherlands	15	100	0	0	15		
			Portugal	11	90.9	9.1	1	10		
			Slovenia	40	100	0	0	40		
			Spain	20	90	10	2	18		
			Sweden	76	98.7	1.3	1	75		
			Switzerland	15	93.3	6.7	1	14		
			Turkey	24	100	0	0	24		
			United Kingdom	43	100	0	0	43		
[21]	Demir *et al*., 2024	2018-2022	Turkey	138	99.3	0.7	1	137	10/10	Low
[26]	Florisson *et al*., 2025	2012-2022	Denmark	116	99.1	0.9	1	115	10/10	Low
[3]	Kierzkowska *et al*., 2025	2014-2023	Poland	25	100	0	0	25	9/10 (1,3-10)	Low

Table S4. Funnel plot analysis identifying publication-level estimates lying outside the 95% funnel boundaries.
Legend: S; susceptible isolates, N; number of isolates, R_count; resistant isolates, TE; transformed effect estimate, seTE; standard error of the transformed effect estimates.

Reference	Author	S	N	R_count	proportion	TE	seTE	funnel_lower	funnel_upper	direction
[37]	Nagy *et al*., 2011	818	822	4	0,99513382	1,49689844	0,0174342	1,426929674	1,495271742	above upper boundary
[48]	Veloo *et al*., 2015	283	283	0	1	1,54110935	0,02969569	1,402897149	1,519304267	above upper boundary
[29]	Jeverica *et al*., 2017	869	869	0	1	1,55384149	0,01695646	1,427866045	1,494335371	above upper boundary
[46]	Sydenham *et al*., 2017	359	359	0	1	1,5444318	0,02637063	1,409414267	1,512787149	above upper boundary
[42]	Sarvari *et al*., 2018	399	400	1	0,9975	1,51047648	0,02498439	1,412131304	1,510070112	above upper boundary
[20]	Cobo *et al*., 2019	77	82	5	0,93902439	1,31070141	0,05504819	1,353206259	1,568995157	below lower boundary
[15]	Badr *et al*., 2020	372	386	14	0,96373057	1,37602918	0,02543286	1,411252296	1,51094912	below lower boundary
[30]	Kierzkowska *et al*., 2020	276	276	0	1	1,54073613	0,03006924	1,402165	1,520036416	above upper boundary
[44]	Soki *et al*., 2020	202	202	0	1	1,53567433	0,03513642	1,392233328	1,529968088	above upper boundary
[23]	Di Bella *et al*., 2022	792	827	35	0,95767836	1,3622311	0,01738145	1,427033066	1,49516835	below lower boundary
[34]	Lõivukene *et al*., 2022	464	483	19	0,96066253	1,36871776	0,02273902	1,416532227	1,505669189	below lower boundary
[39]	Parisio *et al*., 2022	112	129	17	0,86821705	1,19515623	0,04393748	1,374983252	1,547218164	below lower boundary
[16]	Boiten *et al*., 2024	184	184	0	1	1,5340024	0,03681051	1,388952111	1,533249305	above upper boundary

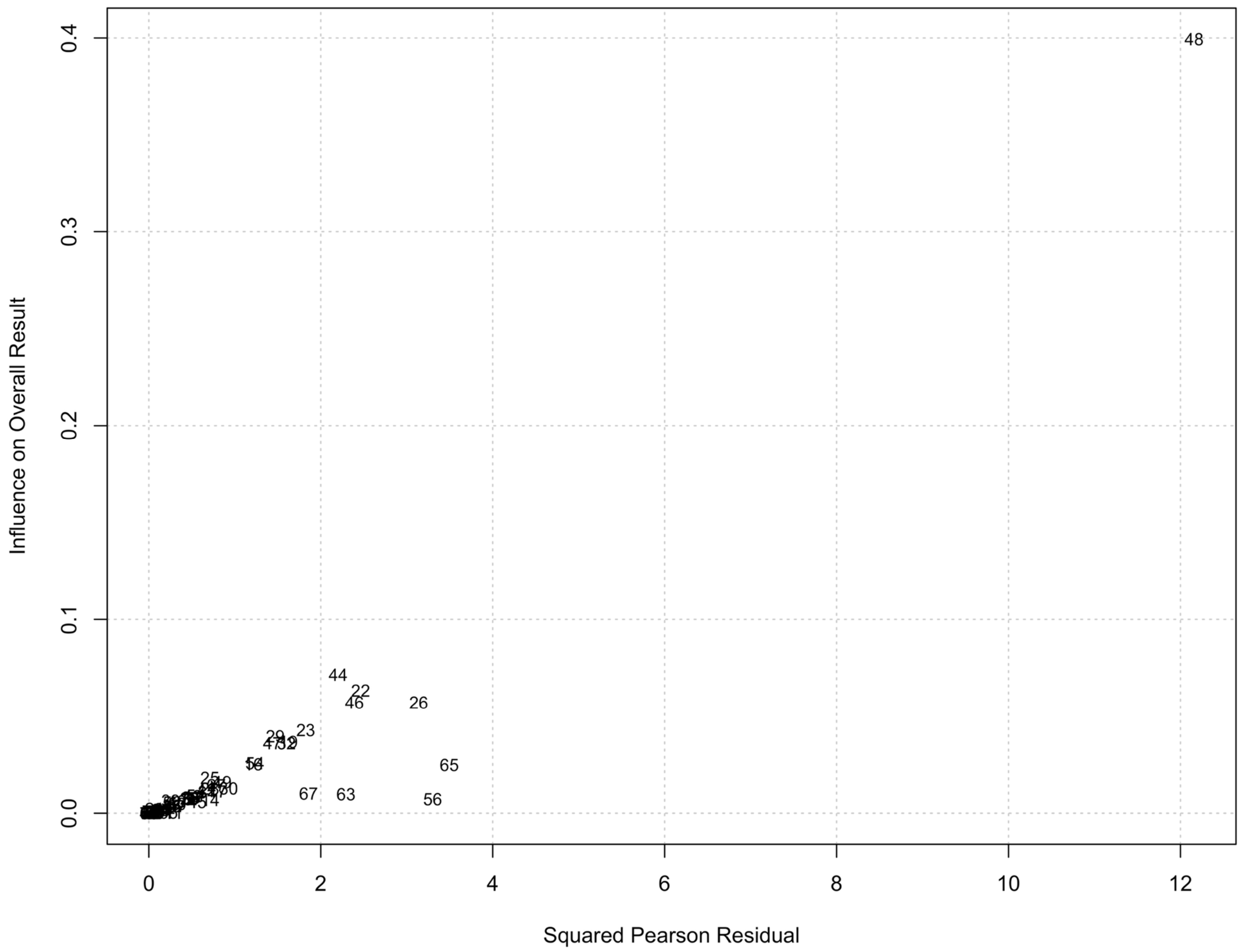

Figure S1. Baujat plot based on publication-level data.

Study | Country	S	N	Proportion	95%-CI	Weight
**Country = Belgium**					
Nagy et al., 2011 | Belgium	103	103	1.00	[0.96; 1.00]	1.7%
Wybo et al., 2014 | Belgium	180	180	1.00	[0.98; 1.00]	2.1%
Soki et al., 2020 | Belgium	40	40	1.00	[0.91; 1.00]	1.1%
Soki et al., 2022 | Belgium	59	59	1.00	[0.94; 1.00]	1.3%
Buhl et al., 2024 | Belgium	4	4	1.00	[0.41; 0.96]	0.2%
**Random effects model**		**386**	**1.00**	**[1.00; 1.00]**	**6.3%**
Heterogeneity: I^2 = 0%, τ^2 = 0, p = 0.9254					
**Country = Croatia**					
Nagy et al., 2011 | Croatia	55	56	0.98	[0.90; 1.00]	1.3%
Novak et al., 2013 | Croatia	34	35	0.97	[0.85; 1.00]	1.0%
Buhl et al., 2024 | Croatia	4	5	0.80	[0.31; 0.95]	0.2%
**Random effects model**		**96**	**0.99**	**[0.95; 1.00]**	**2.5%**
Heterogeneity: I^2 = 22.2%, τ^2 = < 0.0001, p = 0.2766					
**Country = Czech Republic**					
Nagy et al., 2011 | Czech Republic	91	91	1.00	[0.97; 1.00]	1.6%
**Country = Finland**					
Nagy et al., 2011 | Finland	84	85	0.99	[0.94; 1.00]	1.6%
**Country = France**					
Nagy et al., 2011 | France	50	51	0.98	[0.98; 1.00]	1.2%
Dumont et al., 2020 | France	66	67	0.99	[0.92; 1.00]	1.4%
Buhl et al., 2024 | France	79	80	0.99	[0.94; 1.00]	1.5%
**Random effects model**		**198**	**0.99**	**[0.96; 1.00]**	**4.1%**
Heterogeneity: I^2 = 0%, τ^2 = 0, p = 0.9311					
**Country = Germany**					
Nagy et al., 2011 | Germany	72	72	1.00	[0.96; 1.00]	1.5%
Genzel et al., 2014 | Germany	73	73	1.00	[0.95; 1.00]	1.5%
Badr et al., 2020 | Germany	372	386	0.96	[0.94; 0.98]	2.4%
Soki et al., 2020 | Germany	24	24	1.00	[0.85; 1.00]	0.7%
Buhl et al., 2024 | Germany	27	27	1.00	[0.87; 1.00]	0.8%
**Random effects model**		**582**	**1.00**	**[0.97; 1.00]**	**6.9%**
Heterogeneity: I^2 = 53.2%, τ^2 = 0.0034, p = 0.0733					
**Country = Greece**					
Nagy et al., 2011 | Greece	75	75	1.00	[0.95; 1.00]	1.5%

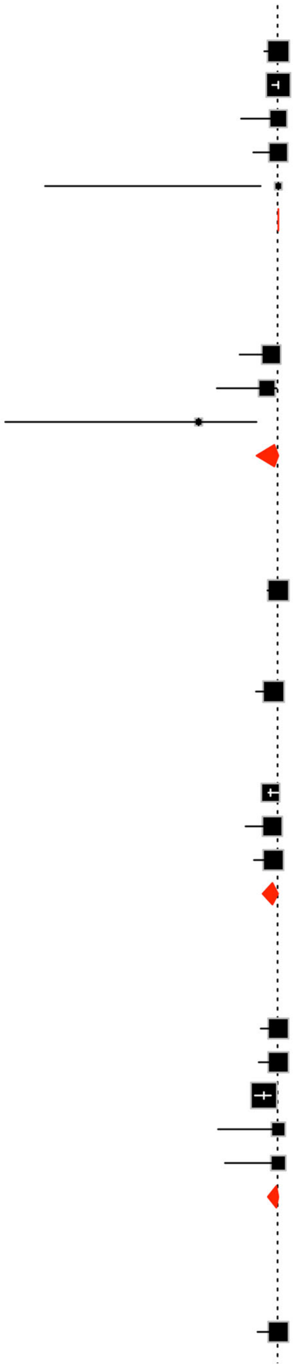

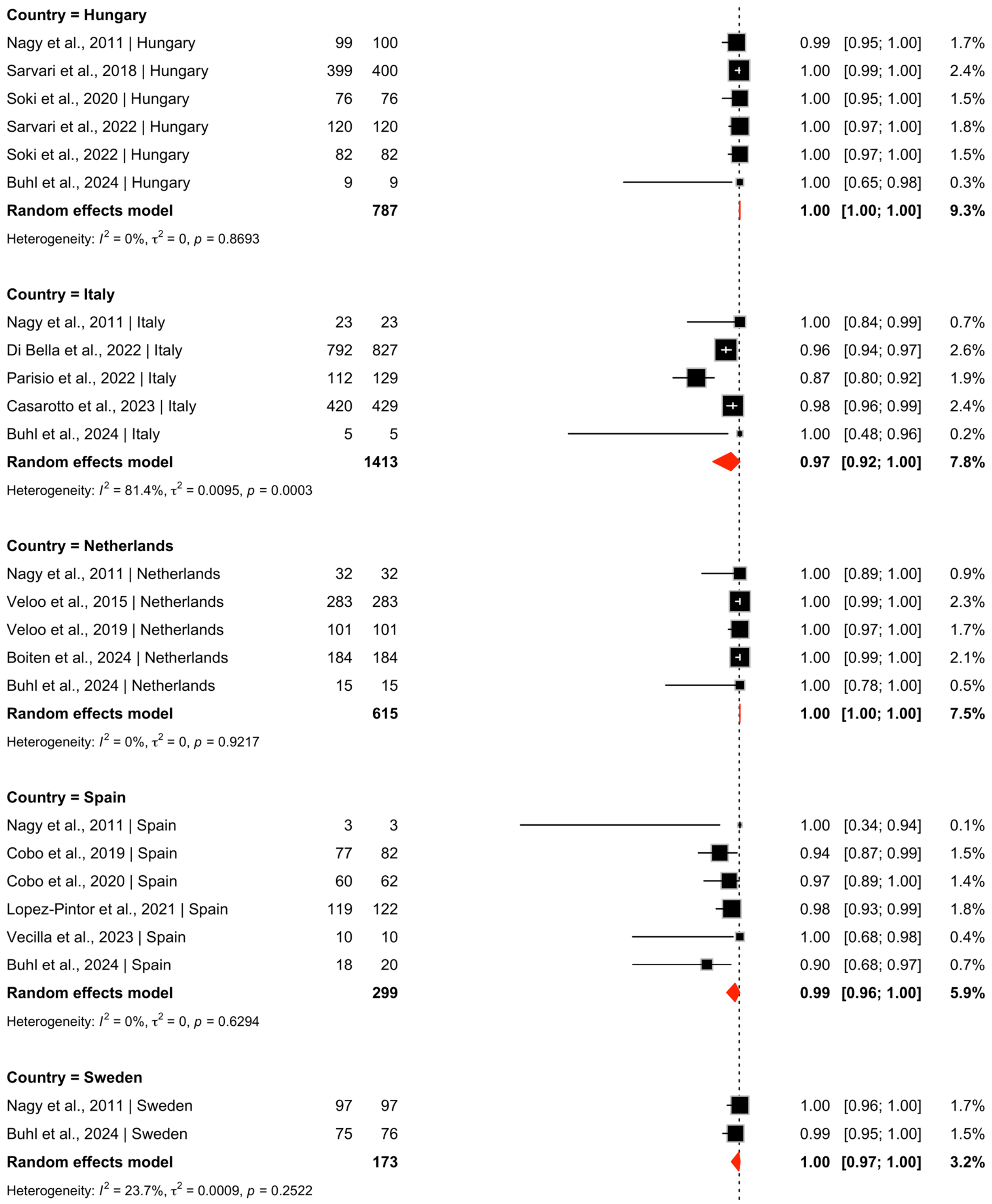
Country = Hungary
Nagy et al., 2011 | Hungary 99 100 0.99 [0.95; 1.00] 1.7%
Sarvari et al., 2018 | Hungary 399 400 1.00 [0.99; 1.00] 2.4%
Soki et al., 2020 | Hungary 76 76 1.00 [0.95; 1.00] 1.5%
Sarvari et al., 2022 | Hungary 120 120 1.00 [0.97; 1.00] 1.8%
Soki et al., 2022 | Hungary 82 82 1.00 [0.97; 1.00] 1.5%
Buhl et al., 2024 | Hungary 9 9 1.00 [0.65; 0.98] 0.3%
Random effects model 787 1.00 [1.00; 1.00] 9.3%
Heterogeneity: I^2 = 0%, τ^2 = 0, p = 0.8693
Country = Italy
Nagy et al., 2011 | Italy 23 23 1.00 [0.84; 0.99] 0.7%
Di Bella et al., 2022 | Italy 792 827 0.96 [0.94; 0.97] 2.6%
Parisio et al., 2022 | Italy 112 129 0.87 [0.80; 0.92] 1.9%
Casarotto et al., 2023 | Italy 420 429 0.98 [0.96; 0.99] 2.4%
Buhl et al., 2024 | Italy 5 5 1.00 [0.48; 0.96] 0.2%
Random effects model 1413 0.97 [0.92; 1.00] 7.8%
Heterogeneity: I^2 = 81.4%, τ^2 = 0.0095, p = 0.0003
Country = Netherlands
Nagy et al., 2011 | Netherlands 32 32 1.00 [0.89; 1.00] 0.9%
Veloo et al., 2015 | Netherlands 283 283 1.00 [0.99; 1.00] 2.3%
Veloo et al., 2019 | Netherlands 101 101 1.00 [0.97; 1.00] 1.7%
Boiten et al., 2024 | Netherlands 184 184 1.00 [0.99; 1.00] 2.1%
Buhl et al., 2024 | Netherlands 15 15 1.00 [0.78; 1.00] 0.5%
Random effects model 615 1.00 [1.00; 1.00] 7.5%
Heterogeneity: I^2 = 0%, τ^2 = 0, p = 0.9217
Country = Spain
Nagy et al., 2011 | Spain 3 3 1.00 [0.34; 0.94] 0.1%
Cobo et al., 2019 | Spain 77 82 0.94 [0.87; 0.99] 1.5%
Cobo et al., 2020 | Spain 60 62 0.97 [0.89; 1.00] 1.4%
Lopez-Pintor et al., 2021 | Spain 119 122 0.98 [0.93; 0.99] 1.8%
Vecilla et al., 2023 | Spain 10 10 1.00 [0.68; 0.98] 0.4%
Buhl et al., 2024 | Spain 18 20 0.90 [0.68; 0.97] 0.7%
Random effects model 299 0.99 [0.96; 1.00] 5.9%
Heterogeneity: I^2 = 0%, τ^2 = 0, p = 0.6294
Country = Sweden
Nagy et al., 2011 | Sweden 97 97 1.00 [0.96; 1.00] 1.7%
Buhl et al., 2024 | Sweden 75 76 0.99 [0.95; 1.00] 1.5%
Random effects model 173 1.00 [0.97; 1.00] 3.2%
Heterogeneity: I^2 = 23.7%, τ^2 = 0.0009, p = 0.2522

**Country = Turkey**					
Nagy et al., 2011 | Turkey	34	34	1.00	[0.89; 0.99]	0.9%
Demirci et al., 2018 | Turkey	28	28	1.00	[0.87; 1.00]	0.8%
Soki et al., 2020 | Turkey	40	40	1.00	[0.90; 1.00]	1.1%
Buhl et al., 2024 | Turkey	24	24	1.00	[0.84; 0.99]	0.7%
Demir et al., 2024 | Turkey	137	138	0.99	[0.96; 1.00]	1.9%
**Random effects model**		**264**	**1.00**	**[0.99; 1.00]**	**5.5%**
Heterogeneity: I^2 = 0%, τ^2 = 0, p = 0.9989					
**Country = Russian Federation**					
Shilnikova et al., 2015 | Russian Federation	65	67	0.97	[0.89; 0.99]	1.4%
**Country = Romania**					
Szekely et al., 2015 | Romania	53	53	1.00	[0.93; 1.00]	1.2%
Pricop et al., 2020 | Romania	75	75	1.00	[0.95; 1.00]	1.5%
**Random effects model**		**128**	**1.00**	**[0.99; 1.00]**	**2.7%**
Heterogeneity: I^2 = 0%, τ^2 = 0, p = 0.9040					
**Country = Poland**					
Kierzkowska et al., 2016 | Poland	74	74	1.00	[0.95; 1.00]	1.5%
Kierzkowska, et al., 2019 | Poland	111	111	1.00	[0.99; 1.00]	1.8%
Kierzkowska et al., 2020 | Poland	276	276	1.00	[0.99; 1.00]	2.3%
Majewska et al., 2021 | Poland	15	15	1.00	[0.82; 1.00]	0.5%
Kierzkowska et al., 2025 | Poland	25	25	1.00	[0.91; 1.00]	0.8%
**Random effects model**		**501**	**1.00**	**[1.00; 1.00]**	**6.8%**
Heterogeneity: I^2 = 0%, τ^2 = 0, p = 0.9095					
**Country = Denmark**					
Hansen et al., 2017 | Denmark	356	359	0.99	[0.98; 1.00]	2.4%
Sydenham et al., 2017 | Denmark	359	359	1.00	[0.99; 1.00]	2.4%
Buhl et al., 2024 | Denmark	50	51	0.98	[0.90; 1.00]	1.2%
Florisson et al., 2025 | Denmark	115	116	0.99	[0.99; 1.00]	1.8%
**Random effects model**		**885**	**1.00**	**[0.98; 1.00]**	**7.8%**
Heterogeneity: I^2 = 54.7%, τ^2 = 0.0015, p = 0.0850					
**Country = Slovenia**					
Jeverica et al., 2017 | Slovenia	869	869	1.00	[1.00; 1.00]	2.6%
Soki et al., 2020 | Slovenia	22	22	1.00	[0.85; 1.00]	0.7%
Buhl et al., 2024 | Slovenia	40	40	1.00	[0.93; 1.00]	1.1%
**Random effects model**		**931**	**1.00**	**[1.00; 1.00]**	**4.4%**
Heterogeneity: I^2 = 0%, τ^2 = 0, p = 0.5422					

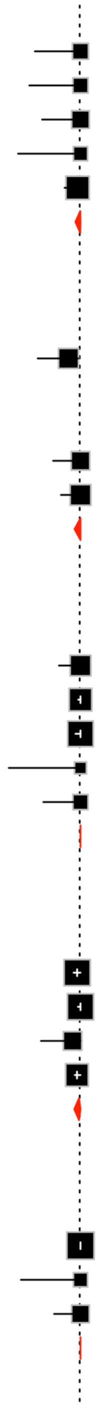

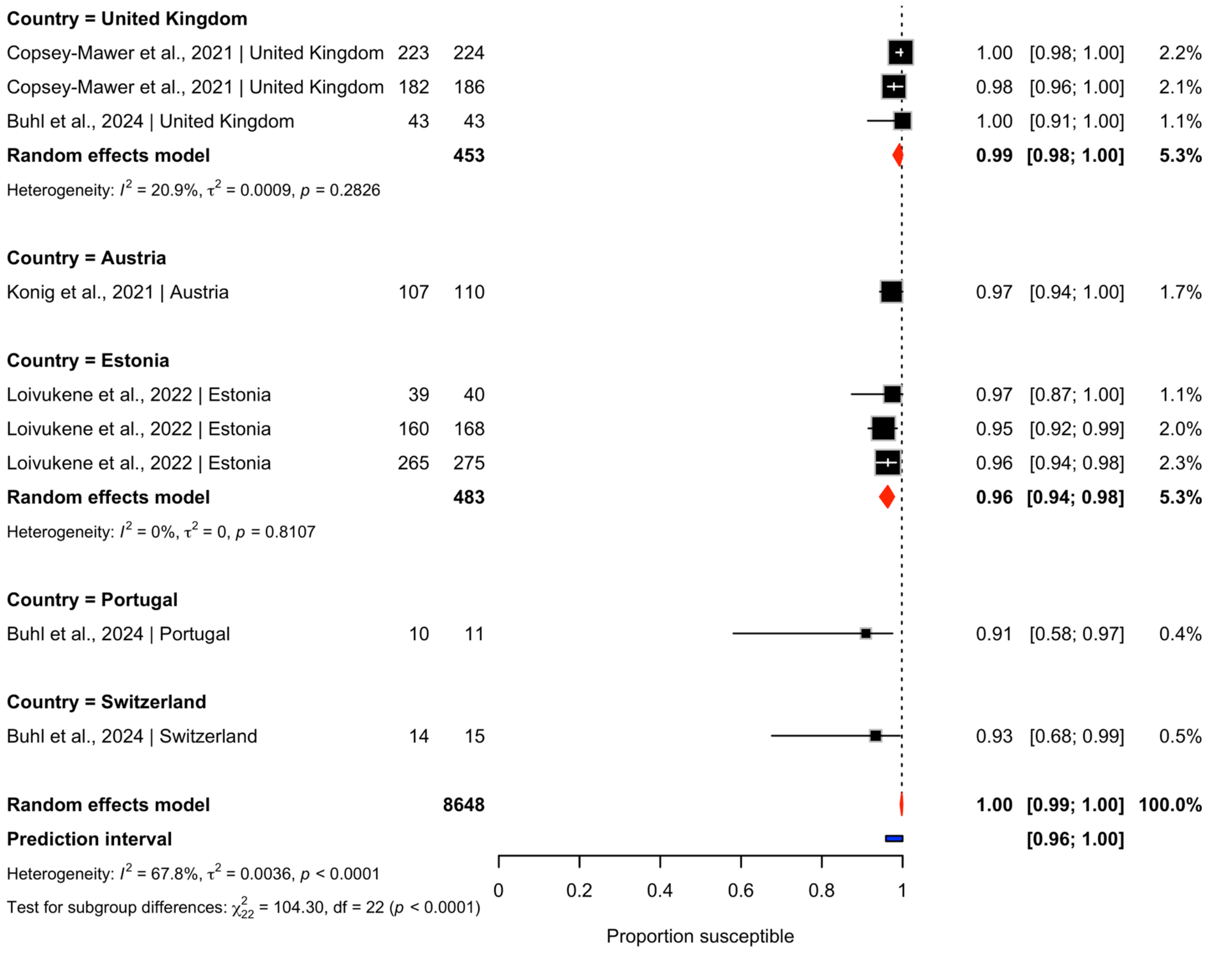

Figure S2. Forest plot of susceptibility proportions by study and country (random-effects model).
